# Supplementary material for: Engineering indel and substitution variants of diverse and ancient enzymes using Graphical Representation of Ancestral Sequence Predictions (GRASP)
Source: PLoS Comput Biol. 2022 Oct 24;18(10):e1010633. doi: 10.1371/journal.pcbi.1010633 (PMC9632902; doi:10.1371/journal.pcbi.1010633)
Supplement: Attachment — Submitted filename: Response 2nd.pdf [file pcbi.1010633.s020.pdf]

Dear Editor/s, Professors Punta and Elofsson, reviewers,

Many thanks for your re-review of our manuscript. We are again grateful for the constructive feedback and recommendations (in black below), which we are in full agreement with. We have responded to each here (in blue) and in the manuscript (tracked in red). We look forward to your decision.

On behalf of all the authors,

Mikael Bodén

**Comments to the Authors:**
**Please note here if the review is uploaded as an attachment.**

Reviewer #2: The revised version of this paper appears to be well updated from the original manuscript according to the comments of the reviewers, including myself. I have written four comments on the original manuscript. The authors seem to have generally responded appropriately to my comments and revised the manuscript accordingly. On my first comment, I think that authors' response satisfies my comments on the alignments and phylogenetic trees which were not mentioned in the original manuscript. On my fourth comment, I apologize for the grammatical problems in my comment. Despite this, I appreciate that the response by the authors makes it easier to understand how much indel (gap/insertion) exists between sequences in the alignments for inferring ancestral sequences in the paper.

However, I would like to comment further on their responses, which are listed below.

On my second comment (In relation to the above section, it is my understanding that only analyses that do not take into account differences in evolutionary rates between loci when estimating ancestral sequences are valid for GRASP. Note that I have also accessed the web page of the authors' web service. I wonder if such an analysis would be able to recover reasonable amino acid sequences and the presence or absence of indels, especially when using data with low sequence conservation and diverse protein subfamilies. I think this also has an impact on the degree to which ancestral sequence recovery can be done with high confidence when using data sets with a high degree of sequence conservation. It seems to me that the limitations of GRASP need to be analyzed/mentioned in this regard.).

I understand authors' policy on the treatment of heterogeneity of evolutionary rate among sites. The addition of statement on p. 29 seems to make better understandings by readers including me. However, the manuscript states that it is possible to use a phylogenetic tree that assumes heterogeneity in evolutionary rates among sites obtained from the IQ-Tree. Does this mean that the branch lengths assuming heterogeneity of evolutionary rates among sites in the phylogenetic tree given are treated the same as the branch lengths of the phylogenetic tree assuming homogeneity of evolutionary rates among sites for the estimation of ancestral sequences and ancestral indels in GRASP?

The reviewer correctly notes that GRASP can only assume that rates are indifferent across sites if none are supplied. If the tree and site-specific rates are *jointly* inferred then these should be provided to the reconstruction for ancestor states to be consistent with that tree—we clarified the relevant statement on page 31.

GRASP itself does not estimate position-specific evolutionary rates[39,40] but can use rates that are inferred jointly with the phylogenetic tree inference by tools such as IQ-TREE 2[41]. In the absence of position-specific rates, GRASP assumes a fixed rate and that the tree is consistent with that.

That said, we specifically assessed the difference between methods that separately reconstructed ancestors with and without position-specific rates and observed that the impact was minor (page 20). This assessment was done for relatively small trees (due to the expense of inferring rates with the reconstruction) but may alleviate a reader's concern about GRASP not enforcing consistency between tree and rates.

Finally, by convention, rates across positions have a mean of 1, and act as a *multiplier* of the branch length (representing time). To be clear, the rates *complement* the branch lengths. It is different for insertions and deletions; they are not influenced by site-specific rates. (In the case of edges in partial-order graphs, indels are associated with at least two sites so GRASP uses branch lengths alone; changes on page 31).

edges that are indeed found in other extants at the same position. We note also that edges are attached to multiple positions and therefore branch lengths dictate how quickly they change, even when positions-specific rates are available.

On my third comment (The authors' analysis of CYP2U/CYP2R/CYP2D discusses the differences in structural stability and substrate specificity of the recovered ancestral proteins due to the presence or absence of three characteristic independent insertions. This in itself is a good analysis to see the effect of the presence or absence of indel by ancestral sequence restoration. However, a large part of the effect of the presence/absence of the insertion sequence on structure and function is likely to come from the difference in 3D structure due to the presence/absence of the insertion sequence structure, since the 3D structure of the CYP2 family is known (e.g. PDBid 2F9Q), and homology modeling, etc. can be used to determine the structure of the CYP2 family. It would be helpful to have a comparison of three-dimensional structures to further discuss the effectiveness of the ancestral sequence reconstruction performed in this program.).
Authors added the comparison of (predicted) 3D structures of ancestral CYP2Us. I am not familiar on the structure of CYP2Us, it is not easy for me to understand the relationship between AA' loop/D-E loop and indels "LLEE" and "LLSPP". Authors are requested to be re-written the paragraph added at p. 12 of revised manuscript to clarify the relationship between AA' loop/D-E loop and indels "LLEE" and "LLSPP".

We have provided further explanation on pages 13-14 and added an additional panel (Panel A) to Supplementary Figure 7.

The major alpha helices in the cytochrome P450 fold are named sequentially from A-L, following the protein backbone[29]. Both insertions discussed here occur within loops in either the AA' loop (LLSPP insertion) or the D-E loop (LSEE insertion) of CYP2U. The AA' loop occurs near the N-terminal region of the protein and is thought to be involved with protein-membrane interactions. The D-E loop is a surface loop and is not located near the structurally important regions, such as the haem binding pocket, the CPR-interaction interface and the putative substrate entry channel[30,31]. Both the AA' loop and the D-E loop are both surface loops positioned on the distal side of the haem cofactor but while the AA' loop is located near the transmembrane helix and is expected to be partially embedded in the membrane, the D-E loop is structurally distant from the AA' loop[30,31] (Supplementary Fig. 7A).

The effects of these ancestral insertions were investigated using models generated using AlphaFold2 run via ColabFold.[32,33] (Supplementary Fig. 7B). However, due to poor confidence in the AA' loop region prediction (Supplementary Fig. 7C-E), the structural models did not yield any additional insights into a role of loop truncation in CYP2U thermal stability. Similarly, only minor changes to the D-E loop were observed for the ancestors bearing the LSEE insertion (Supplementary Fig. 7F).

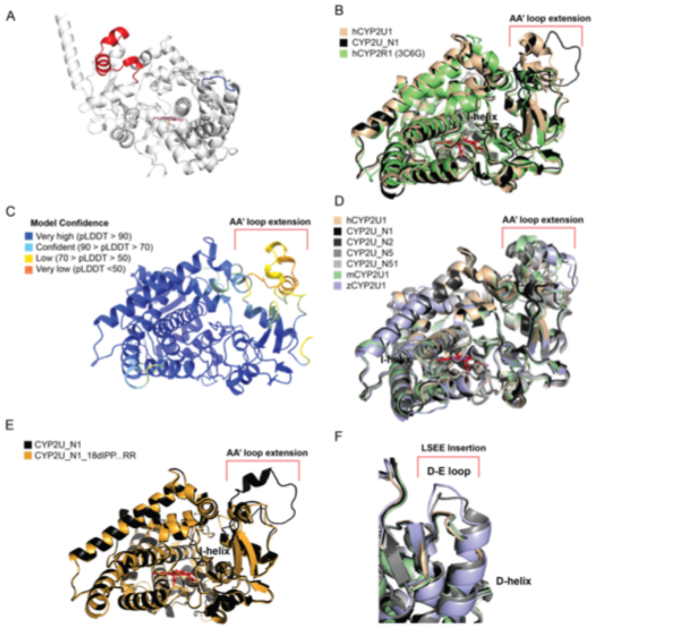


Figure 7: AlphaFold2 models of CYP2U ancestral and extant proteins.**a**, AlphaFold2 model of human CYP2U1. The AA' loop is shown in red, the D-E loop is shown in blue, and the haem cofactor is shown in pink. **b**, Structural alignment of the AlphaFold2 models for human CYP2U1 (beige) and CYP2U_N1 (black) with the human PDB structure for CYP2R1 (green), which is the closest relative of CYP2U1 with an experimental structure. CYP2R1, like all other CYP2s, lacks the AA' loop extension found in CYP2U proteins. The haem cofactor is depicted in red. **c**, AlphaFold2 structure for human CYP2U1 showing the low model confidence for the AA' loop extension. **d**, Structural alignment of the AlphaFold2 models for all the CYP2U ancestors and three extant CYP2U proteins (human CYP2U1, mouse CYP2U1, zebrafish CYP2U1), showing the differences in the predicted positioning of the AA' loop extension. The haem cofactor is shown in red. **e**, Structural alignment of the AlphaFold2 models for the CYP2U_N1 ancestors with and without the AA' loop insertion. Aside from the AA' loop, no substantial structural differences are observed between these structures. **f**, Structural alignment of the AlphaFold2 models for all the CYP2U ancestors and three extant CYP2U proteins (human CYP2U1, mouse CYP2U1, zebrafish CYP2U1), showing the elongation of the D-helix and D-E loop in the zebrafish CYP2U1 (blue) and CYP2U_N5 (grey) with LSEE insertion.

Reviewer #3: Again, I used the same two expert sub-reviewers, one with an algorithmic bent and the second with a protein and structural bent. Their reports are presented below. Both acknowledge the authors' efforts to improve the clarity and address all the referees' points. However, they still have many comments and concerns. They recommend acceptance with minor (but mandatory) changes, and I agree with this opinion. Hope this helps!

********************** First sub-reviewer **********************

The main comment I had about this manuscript was about the clarity of the new algorithm. Both reviewer 1 and the editor also noted that this had to be improved. Because the authors appear to have put some effort into this, I had another good look into this. Here are two (hopefully constructive) remarks:

A) First, the description of the bi-directional approach still sounds more complicated than needed. If I had to describe it in my own words I would have said something like:

For any given internal node k of the tree, and for any site i, the goal is to infer two things:
- the right neighbor of site i in the sequence at k,
- the left neighbor of site i in the sequence at k.

These can be viewed as two characters that can be separately inferred via standard methods in phylogenetics (max parsimony and likelihood), on the basis of the right/left neighborhood relationships in the observed (extant) sequences.

Barring one detail (see next point), this is probably all that a specialist of ancestral reconstruction will need to understand the bi-directional approach, which is the only methodological novelty here.

We are grateful for the reviewer's suggestion and took inspiration from it to update the preliminary description of bi-directional edge methods (page 7). As a result we made further clarifications on that same page, as well as providing an additional perspective in the more technical description (page 29).

GRASP enumerates all edges in $\mathbf{E}*$ and infers $\mathbf{E}^{(k)}$ at each ancestor $k$ by either *maximum parsimony* or *maximum likelihood*. This inference involves solving multiple independent optimisation problems, avoiding scalability issues of earlier approaches[12,13]. GRASP assigns scores (for parsimony) or probabilities (for maximum likelihood) to (combinations of) edges leaving and edges entering each reference position $i$. In other words, the goal is to infer for each position in an ancestor the best left and right neighbours. In the simplest case, a position in an ancestor has a single left neighbour and single right neighbour and this resolves to a linear sequence. In cases where a position has more than two neighbours, we benefit from representing these alternative neighbours as paths in a POG. This means that each edge is in fact evaluated twice: once in the forward direction, once in the backward direction. To formally distinguish the two evaluations, we refer to $(a, b)$ and $(b, a)$, where $a$ and $b$ are topologically sorted position indices; in other words, the diagonal of the adjacency matrix distinguishes forward from backward edges.

B) A point where there remains some ambiguity is the following, and I really believe that this needs to be clarified:

When site i is not present in an extant sequence (ie. at a leaf of the tree), how do we set the characters encoding its left and right neighbor? There are two options:
- we treat them as missing data, which both parsimony and likelihood know how to deal with;

- we treat them as a special character competing with the other possible left/right neighbors. In the revised manuscript the authors say that they have chosen this option. They call this the "no edge" option.

What worries me is that the behavior of the online tool seems to contradict the claims made in the manuscript. All the tests I made at http://grasp.scmb.uq.edu.au/ are consistent with encoding the characters above as missing data, not as "no edge"!

The reviewer is correctly observing that the implementation of the web service (which they used) is behind that of the command line tool (which we describe). We believe the "no-edge" option is a principled approach that we expect to incorporate across the suite of tools. Since the update of the web service is an undertaking that we need more time for, we are instead providing a "legacy" option in the command line tool so that reconstructions of indels can be reproduced regardless of the original version. What the legacy version does is to clip the tree from below to remove the index from an ancestor if all of its children miss it. Then parsimony proceeds to assign edges between indices that remain. This works analogously with maximum likelihood. We made these important clarifications on page 30.

The equations define the implementation of bi-directional edge parsimony in GRASP, but one special case warrants attention; when one or more extant sequences is missing the reference position ($a$ in Eq. 3 or $b$ in Eq. 4) a "no edge" state can be added to the total set of edges considered and thus compete with normal edges at ancestors. This option is currently available through the command line implementation of GRASP. To enhance readability, we have refrained from including it in the equations and matrices. The legacy approach (used for all experimentally validated predictions in this manuscript) is to (a) exclude the reference position at ancestors for which *all* extants are missing, and (b) infer a value for all remaining ancestors selected from those seen in other extants.

We have further clarified the distinction between the versions on page 35.

Additional information and a suite of tools to assist in the application of GRASP are available at `https://bodenlab.github.io/GRASP-suite`. An implementation of bi-directional edge parsimony indel inference that considers gaps as missing data rather than explicitly encoding gaps as an additional "no edge" state is available through the web server. All other indel inference methods as well as the option to either consider gaps as missing data or additional states are available through a separate command line version. The implementation in Java and a web application are available from the same site. The software is available under the GNU General Public License v3.0.

To make sure that the web service is used appropriately while we work on its update, we have added a note to the web page.

In particular the example in Fig. 6 (introduced in the revised manuscript), allows one to discriminate between the two options above. The online tool launched on this example returns a POG for node N1 where the edges (2,3) and (3,4) have bi-directional support. This is consistent with the "missing data" encoding. However it contradicts the diagrams shown in Fig. 6, where the two edges cited above only have uni-directional support, which is instead consistent with the "no edge" encoding.

(The reason for these observations are technical but easy to understand: site 3 is only observed in one of the 5 input sequences, so its left and right neighbor at node N1 will be assigned the "no edge" option if it's available; however if the neighbors of 3 are treated as missing data, they will be assigned the only observed left and right neighbors, which are 2 and 4 respectively.)

The source of the contradiction above is not due to the fact that the online tool implements max likelihood, while Fig. 6 is based on parsimony: I made sure to provide a tree with constant and very small branch lengths, where likelihood and parsimony are equivalent. Here are the file contents necessary to reproduce this example:

- The tree: ((a:0.001,b:0.001):0.001,c:0.001,(d:0.001,e:0.001):0.001);
- The sequence alignment:

```
>a
CS-PW
>b
RS-PW
>c
MADPW
>d
MA--W
>e
MA--W
```

To enable users to explore the impact of the “no edge” option, we have implemented functionality in the command line tool to turn it off; the example in Fig. 6 gives two subtly different outputs as shown here:

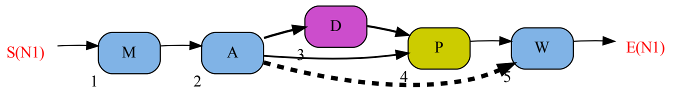


*N1 when gaps imply “missing data”.*

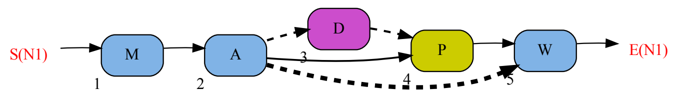


*N1 when gaps imply "no-edges".*

Minor comments
--------------

- The superscripts of E in equations (3) and (4) should be (c), not (j).

- In Fig. 6, E^N2(2,5) should be 0, not 1.

We thank the reviewers for their very thorough review of the text and their constructive feedback, which we now acknowledge in the manuscript. We have now fixed these typos.

********************** Second sub-reviewer **********************

Thanks to the authors for their detailed responses. I appreciate the work they have done trying to address any concerns and questions — including those questions that I asked out of simple curiosity. Next, you will find my comments on each of the authors' answers:

(1) Thanks for clarifying the decision behind choosing MAFFT. I have found it interesting that you have found no further improvements in the accuracy of the INDELible MSA reconstructions using ProGraphMSA.

(2) [A] I understand the point made by the authors. Small changes can indeed cause functional changes and have a lesser structural impact. However, it would be better to assess the structural impact of large indels rather than simply assume them to be destabilizing or artifacts. For example, the AA' loop, a region longer than 20 residues, doesn't look essential for the overall domain fold. In fact, it is located between the transmembrane region — or included in the membrane — and the PF00067 domain. Note that even single domain proteins can have long embellishment outside the conserved structural core that can come from insertions or deletions events through evolution. In any case, the 20 residue cutoff for CYP2U1 could be acceptable as the fold is relatively rigid. [B] That's great. I wonder how common those overlaps are in the presented data. It could be great to add the distribution of indel lengths into supplementary materials. That would help the reader understand the indel length range on which you tested the software.

We agree and have added these distributions as a new Supplementary Fig. 2. They further the case we made from Supplementary Fig. 1.

(3) Thank you so much for performing this analysis; the results are interesting, even if they

are not decisive. I favor not including ENST00000508453.1. I also agree with the author's decision after looking at some orthologs; 5 to 6 exons sound fine for them. However, filtering everything by the number of exon changes sounds arbitrary. But, I understand that it is not easy to have quality assessments, such as the ones described for CYP2U1, for non-model species.

Yes; it has been left as is.

(4) Thanks for clarifying. Could you split that sentence into two to ease the reader's work? It's not clear how the sequences have been processed. In particular, have they been processed by trimAl?

We have tried to clarified this on pages 35-36.

the alignment trimmed for positions with >99% gaps by trimAl v1.2[50]. The alignment was pruned using Gblocks 0.91b[51] with a less stringent block selection and the phylogenetic tree was inferred by PhyML[52] using default settings, with subtree pruning and regrafting to optimise tree topology, smart model selection, and aLRT SH-like branch support calculated. The tree was rooted on the midpoint. The full alignment, trimmed by trimAl and with a trimmed N- and C-termini but not pruned by Gblocks was used to perform a marginal reconstruction of ancestral nodes with the LG evolutionary rate model[45], with indels inferred using bi-directional edge parsimony.

(5) Thanks for adding the AlphaFold2 structures; it helps to have an idea of the structural location and impact of the indels.

(6) Thanks for the clarification. I think the procedure is ok; I was curious. Now, it is unclear how the sampling was performed to avoid potential bias from the phylogenetic signal. I guess they would never be independent samples given their homology.

We agree that there is still an underlying phylogenetic signal common across all the trees due to their homology, but feel that re-inferring multiple trees rather than inferring one large tree and pruning it still mitigates some of the influence that a single tree may have over the process.

(7) Thanks for clarifying this; sadly, it is still unclear to me which variables are used to calculate the entropies. Could you please add the equations? I think that could make it more clear.

We hope the revised description on page 42 is clear.

To objectively identify such outliers, we determined the proportion of gaps in each column (number of sequences with gaps in given column / number of sequences), and used this to calculate the Shannon entropy for a given sequence at each position (entropy of either gap or amino acid, as found at the position). The position-specific entropies were then summed across each aligned sequence, for all sequences. This demarcated approximately the top 1% of sequences due to their atypical gap patterns, which we decided to remove, resulting in a final set of 11,756 sequences.
