# Supplementary material for: Engineering indel and substitution variants of diverse and ancient enzymes using Graphical Representation of Ancestral Sequence Predictions (GRASP)
Source: PLoS Comput Biol. 2022 Oct 24;18(10):e1010633. doi: 10.1371/journal.pcbi.1010633 (PMC9632902; doi:10.1371/journal.pcbi.1010633)
Supplement: Attachment — Submitted filename: Response 3rd.pdf [file pcbi.1010633.s021.pdf]

Dear Editor/s, Professor Elofsson,

Many thanks for your re-review, which we have included in full below. As a result, we have improved the legibility (and quality in general) of all figures in the manuscript, including some of the figures in the supplement.

On behalf of all the authors,

Mikael Bodén

Editor's Comments:

Please note the comment from one reviewer: I would like to comment in the sense that I would like the paper to be easy to read. That is, in some figures, the letters used in the figures are too small and difficult to read. For example, the letters in the graphs c), d), and e) in Figure 3 seem too small to read.

Reviewer's Responses to Questions

Comments to the Authors:

Reviewer #2: In the revised version of this paper, authors changed parts where I (and other reviewers) commented to be reconsidered. I think that most of them are improved the clearness of this paper. I think this paper is useful for researchers who are doing ancestral sequence resurrection.
I would like to comment in the sense that I would like the paper to be easy to read. That is, in some figures, the letters used in the figures are too small and difficult to read. For example, the letters in the graphs c), d), and e) in Figure 3 seem too small to read.

Reviewer #3: Thanks to the authors for addressing all my comments
