# Supplementary material for: Engineering indel and substitution variants of diverse and ancient enzymes using Graphical Representation of Ancestral Sequence Predictions (GRASP)
Source: PLoS Comput Biol. 2022 Oct 24;18(10):e1010633. doi: 10.1371/journal.pcbi.1010633 (PMC9632902; doi:10.1371/journal.pcbi.1010633)
Supplement: Attachment — Submitted filename: Response.docx [file pcbi.1010633.s019.docx]

"Engineering indel and substitution variants of diverse and ancient enzymes using Graphical Representation of Ancestral Sequence Predictions (GRASP)" for consideration at PLOS Computational Biology

**Response to reviewers**

We are very grateful to the editor, the three main reviewers and two sub-reviewers for their detailed feedback and constructive suggestions, which have genuinely improved the manuscript. We have considered and responded to each point, and in most instances made changes to the manuscript. We hope that you find the changes satisfactory. In summary, we have undertaken a substantial reworking of the description of the main method, added a new figure (Figure 6) to assist the understanding of the indel inference, and made clarifications regarding sequence data and experimental steps taken when our presentation was lacking.

We have included the qualified decision from the editor, and all reviewer comments verbatim, with our responses and pointers to the changes that have been made as a result highlighted in blue font (red font when copied directly from the revised manuscript). Substantively revised text has been highlighted in red font in the manuscript, with minor changes including typos *not* highlighted to improve readability.

Thank you.

Mikael Bodén on behalf of all authors

**Editor decision**:

In light of the reviews (below this email), we would like to invite the resubmission of a significantly-revised version that takes into account the reviewers' comments.

Besides addressing all other comments from the reviewers, please make every effort to render the algorithm at the basis of your method clear and understandable.

We have reworked the description of the method and how it is communicated in the manuscript. We have addressed all other concerns individually, as presented below under each reviewer comment.

**Reviewer #1**: This study aims to provide a new way to treat indel events for ancestral sequence reconstruction. This is an important problem for this field and better methods would be very welcome. Unfortunately I am in no position to evaluate this manuscript. I could not follow the mathematical descriptions of what this method does exactly. This is not meant as a judgment of the authors' work; this reviewer was simply to stupid to understand the method. I would suggest, however, that the authors describe the method in the main text and accompany it with figures that explain how it works. As it stands, the new method is only described in the methods section.

We have incorporated multiple changes as suggested by this reviewer and other reviewers in subsequent sections in this document. This includes a new Figure 6 (p. 26), some amendments to describe the method in the main manuscript (p. 5) and major updates in the Methods section (p. 22-28).

**Reviewer #2**: This paper introduces a program called GRASP, which performs Indel estimation in ancestral sequence estimation, shows its applications, and attempts to demonstrate the usefulness of this program. Ancestral sequence reconstructions have become widely used in the research fields of molecular evolutionary biology and protein engineering. In this context, the accurate estimation of indels is important for the creation of proteins that actually have the "correct" structure and functionality to be analyzed based on the estimated sequence.
　GRASP, presented in this paper, can infer ancestral sequences including Indel in six different ways, given an alignment and phylogenetic tree. It is important that the user can choose the method of analysis. In addition, the authors actually used this program to perform ancestral sequence estimation on data from different protein families, and actually produced some of the ancestral proteins for functional analysis. In addition, the authors have also performed functional analysis of proteins based on the ancestral sequence estimation results based on different data sizes (number of sequences in the alignment/phylogenetic tree) for each protein family. The results of these analyses suggest that GRASP's ancestral sequence estimation, including Indel, is relatively robust to differences in protein family types and analysis data sizes. This should be useful for researchers who perform many ancestral sequence reconstructions.
　However, we have the following questions and comments about this paper.

1 The authors have performed ancestral sequence reconstruction experiments, including actual ancestral protein production using GRASP, for three protein families (CYP2U/CYP2R/CYP2D, DHAD, and KARI). As the basal data, the extent to which the sequence data for each of the present organisms falls within the range of sequence similarity should be clearly indicated. In addition, the readers should be able to access the actual alignment, phylogenetic tree, and expressed and analyzed protein sequences. This will allow the readers to better judge the validity of the authors' analyses.

This lack of data set summary was an oversight on our part and we thank the reviewer for highlighting the importance. We have included a new Supplementary Fig. 16 which shows the mean percent identity between extant sequences and a single, nominated ancestral sequence (the root node, N0, in all data sets except for the GDH-GOx, which was a marginal reconstruction performed at N320 and so for this data set we have chosen N320 as the nominated ancestor sequence). The figure quantifies sequence identity across the experimental data sets evaluated within the manuscript and confirms that they probe a wide range of sequence diversity.

Changes have been incorporated into a new section Data availability on p. 33, and refers (in turn) to Supplementary Fig. 16 (also included below).

**Supplementary Fig. 16**: Mean sequence identity between extant sequences and a selected ancestral node. Positions to compare are identified from the multiple sequence alignment for each data set. The selected ancestral sequence is N0, the root node, from a joint reconstruction for each data set except for GDH-GOx, in which the marginal reconstruction of N320 is the selected ancestral sequence.

2 In relation to the above section, it is my understanding that only analyses that do not take into account differences in evolutionary rates between loci when estimating ancestral sequences are valid for GRASP. Note that I have also accessed the web page of the authors' web service. I wonder if such an analysis would be able to recover reasonable amino acid sequences and the presence or absence of indels, especially when using data with low sequence conservation and diverse protein subfamilies. I think this also has an impact on the degree to which ancestral sequence recovery can be done with high confidence when using data sets with a high degree of sequence conservation. It seems to me that the limitations of GRASP need to be analyzed/mentioned in this regard.

This is a fair point. A user can perform a reconstruction that uses position specific evolutionary rates with GRASP; this has now been clarified in the manuscript under the section Inferring the character state of ancestor nodes on p. 29. The paper does not evaluate this feature, since the tool itself does not estimate such variable rates across positions. GRASP implements functionality that enables the provision of user-supplied rates, as generated by phylogenetic tree inference tools. This does not influence inference of indels as both fixed and variable rates will have a mean of 1 across the sequence, and indels are generally not designated a single position; they would involve at least two so currently GRASP will not attempt to use provided evolutionary rates for indel inference.

3 The authors' analysis of CYP2U/CYP2R/CYP2D discusses the differences in structural stability and substrate specificity of the recovered ancestral proteins due to the presence or absence of three characteristic independent insertions. This in itself is a good analysis to see the effect of the presence or absence of indel by ancestral sequence restoration. However, a large part of the effect of the presence/absence of the insertion sequence on structure and function is likely to come from the difference in 3D structure due to the presence/absence of the insertion sequence structure, since the 3D structure of the CYP2 family is known (e.g. PDBid 2F9Q), and homology modeling, etc. can be used to determine the structure of the CYP2 family. It would be helpful to have a comparison of three-dimensional structures to further discuss the effectiveness of the ancestral sequence reconstruction performed in this program.

This comment is related to that of Reviewer 3/Second sub-reviewer (below) and we opted to respond to both here. We had originally modelled the ancestral sequences, but as this analysis yielded no structural insights of note, we omitted it from the original manuscript. As requested by the reviewers, we have now provided AlphaFold2 models for the CYP2U ancestors and three extant CYP2U1 proteins (human CYP2U1, mouse CYP2U1 and zebrafish CYP2U1) and contrasted the structures in the new Supplementary Figure 6. It is worth noting that although experimental structures are available for human CYP2D6 and human CYP2R1, which are the closest relatives to the CYP2U subfamily, these proteins only share 40.2 % (CYP2D6) and 40 % (CYP2R1) percent identity to the human CYP2U1. Furthermore, both CYP2D6 and CYP2R1 completely lack the AA’ loop insertion in the CYP2U1 sequence, which is only poorly modelled in the homology model structures, possibly due to its hypothesised role in membrane binding (Ducassou et al., 2017).

As shown in the included figures, when the AlphaFold2 structures were generated for the CYP2U ancestors, the structures of all proteins closely resembled each other, aside from the positioning of the two loop regions where the insertions are predicted to occur (the D-E loop for the fish insertion) and the AA’ loop, where the structure is poorly modelled. Because no PDB structure in the database contains the AA’ loop, AlphaFold2, which relies on PDB structures for its analysis, is unlikely to yield a reliable structure for this region. Interestingly, the putative substrate entrance channel (Ducassou et al., 2015, 2017) is present in all the CYP2U ancestor models. The AA’ loop varies in length between the inferred ancestors. If, as predicted for human CYP2U1, this loop is embedded in the membrane (Ducassou et al., 2017) and if it acts as a membrane anchor, the structure of this loop may vary substantially without significantly affecting its function.

We did not perform this structural analysis on the DHAD sequences as the CYP2U sequences were the ones within this study that were specifically modified through incorporation of indel events and so, as suggested by the reviewers, it was important to highlight where these modifications were actually present within the structures.

NOTE:

Docassou et al. 2015 - <https://doi.org/10.1016/j.bbagen.2015.03.014>

Ducassou et al. 2017 - <https://doi.org/10.1016/j.biochi.2017.07.007>

We made the following changes to the manuscript on p. 12 and included a new Supplementary Fig. 6:

Sequence changes were introduced in either the AA’ loop or the D-E loop of CYP2U and these regions were investigated using models generated using AlphaFold2 run via ColabFold (Supplementary Fig. 6A). However, due to poor confidence in the AA’ loop region prediction (Supplementary Fig. 6B-D), the structural models did not yield any additional insights into a role of loop truncation in thermal stability. Similarly, only minor changes to the D-E loop were observed for the ancestors bearing the fish insertion (Supplementary Fig. 6E).

**Supplementary Fig. 6**: AlphaFold2 models of CYP2U ancestral and extant proteins. **a,** Structural alignment of the AlphaFold2 models for human CYP2U1 (beige) and CYP2U_N1 (black) with the human PDB structure for CYP2R1 (green), which is the closest relative of CYP2U1 with an experimental structure. CYP2R1, like all other CYP2s, lacks the AA’ loop extension found in CYP2U proteins. The haem cofactor is depicted in red. **b,** AlphaFold2 structure for human CYP2U1 showing the low model confidence for the AA’ loop extension.  **C**, Structural alignment of the AlphaFold2 models for all the CYP2U ancestors and three extant CYP2U proteins (human CYP2U1, mouse CYP2U1, zebrafish CYP2U1), showing the differences in the predicted positioning of the AA’ loop extension. The haem cofactor is shown in red. **d**, Structural alignment of the AlphaFold2 models for the CYP2U_N1 ancestors with and without the AA’ loop insertion. Aside from the AA’ loop, no substantial structural differences are observed between these structures. **e**, Structural alignment of the AlphaFold2 models for all the CYP2U ancestors and three extant CYP2U proteins (human CYP2U1, mouse CYP2U1, zebrafish CYP2U1), showing the elongation of the D-helix and D-E loop in the zebrafish CYP2U1 (blue) and CYP2U_N5 (grey) with LSEE insertion.

4 The authors restrict their analysis of data with a high rate of assumed Indels to just However, from a protein engineering perspective, it would be desirable to be able to express and prepare soluble/analyzable ancestor proteins from data with a high percentage of Indel, which can also be described as low sequence similarity. This has advantages, such as increasing the likelihood that enzyme proteins with lower substrate specificity can be made. Real data on such analysis would be very good. Reanalysis of existing data (e.g., reanalysis of ancestral sequence reconstructions from other groups using GRASP) is also acceptable.

We were unable to find existing data that would meet the requirement of having high indel events, but we do note that the existing data within this paper does not have low indel rates, and that the larger reconstructions have gap percentages that place them between the first and second indel rates studied in the simulated data (where methods were still performing well on simulated data). These facts were obscured by 1) our lack of easily making the data available to access as well as 2) lack of summary information about gaps in the datasets, including the simulated data. To this end, we have included all the data within a repository (explained in a later comment) but, more to this point, we now provide a new Supplementary Fig. 1, that highlights the percentage of gaps in each reconstruction, with the 9,112 DHAD reconstruction reaching an overall gap percentage of ~60% and a remark in the manuscript (p. 7) that highlights that the simulated data is parameterised to create alignments that are comparable to or contain more gaps than the real data.

**Supplementary Fig. 1:** Percentage of positions across every sequence in a given alignment that contains a gap character, for the nine data sets, the simulated data from INDELible, and the simulated data from INDELible as realigned by MAFFT. Simulated data is shown for 500 extant sequences at indel rates of 0.001, 0.005, 0.01, and 0.03.

**Reviewer #3**: I asked two expert sub-reviewers, one with an algorithmic bent and the second with a protein and structural bent. Their reports are presented below. Both found the work important and interesting, with remarkably well-made software. However, the former was not able to understand the details of the algorithms, and the latter has several comments, particularly on the length of the indels. Thus, I (we) recommend acceptance pending major revisions, corresponding to the points below. Hope this helps!

**First sub-reviewer**:
From an algorithmic standpoint, the main contribution of this manuscript is the proposal of a new graph-based method to deal with insertions and deletions in ancestral sequence reconstruction (ASR). The new method is named BE (bidirectional-edge encoding) and is implemented in the GRASP software. GRASP is compared against:

- PS (single-site position-specific reconstruction) implemented in PAML, and

- SIC (simple indel encoding) implemented in FastML.

GRASP is capable of handling large trees, and produces reconstructions similar to those of FastML, with the added advantage of presenting them with a graph formalism, which is the natural solution to represent uncertainty in the reconstruction.

I believe that the GRASP software will be a very useful tool for ASR (I played a bit with the tool at <http://grasp.scmb.uq.edu.au/> and I'm very positively impressed). However, I have a few comments on the algorithmic side of this work. These are easy to address, although they will require some more writing:

**** (1) ****
I have tried really hard to understand the details of the algorithm at page 32, but I cannot say I understand it. This is important, as this algorithm forms the basis of the new methodology presented here.

We found the comments below to be extraordinarily helpful and we have made sweeping changes accordingly (some already highlighted under Reviewer 1). Specifically, we have improved the explanation of the novel elements of indel inference. This includes the calculation of support that draws reference between the reworked, precise mathematical formulation and a new figure that highlights the effect of calculating support for indels by means of an example.

Here are the aspects that are unclear:
** 1a. About binary matrix E representing the POG, defined at lines 782-790: Is this matrix triangular? That is E(a,b)>0 only for pairs a

For the input POG and extant POGs, all the entries below the main diagonal are zero, as these matrices are used to specify existence of an edge between two nodes. Based on sub-reviewer 1’s suggestion 1d below, we now use the ancestor POG matrices to indicate support for a given edge in the forwards (above the diagonal) and backwards (below the diagonal) directions, and so both upper and lower entries may contain values.

The updated indexing nomenclature of *(a,b)* and *(b,a)* refers to the upper and lower entries within a matrix, and changes have been made throughout the text starting on p. 23:

Nodes are connected by a set of directed edges, which is conveniently represented by a so-called adjacency matrix **E**, where **E**(*a, b*) is set to 1 if there is an edge from *a* to *b*, and otherwise 0; *a* is topologically *before b*, meaning that a *uni-directional* graph can be represented by above-diagonal elements in **E**.

Figure 6 also indicates that an input POG is only informative above the main diagonal, and highlights how the pairing of *(a,b)* and *(b,a)* positions above and below the diagonal in the ancestor matrices allow for support to be either uni-directional or bi-directional.

** 1b. About equation (1) at line 835:
- The equation would benefit from being decomposed into several equations. It is very hard to read as is.

We have now split equation (1) into equations (1), (2), (3), (4), and (5) (see p. 27-28) to more clearly distinguish the forward and backward edge calculations as well as the specific cost function used when calculating parsimony scores, which is recursively defined still.

- When c belongs to J (i.e. c is a leaf, rightmost cases in lines 1 and 3), the added cost is 0, but this sounds wrong to me: this appears to imply that the cost of any edge at a leaf is 0, but this sounds wrong. Only edges in the extant POG E^(j) should receive a cost of 0, while the remaining ones should have a positive cost (1 or infinite?)
- In fact, sigma(j,delta,a,b) for j belonging to J (a leaf) should be explicitly defined somewhere, as it will constitute the base case of the recursion.

Exceptional catch. This was indeed an error on our part in the formulation of the equation and it is now corrected in the manuscript. Furthermore, we now directly reference the cost function, and the base case is now explicitly defined within the text of the manuscript, with references to where it fits into the formulation.

** 1c. The sentence "the edge with best score [...] is assigned a score of 1" is confusing (line 831). I guess that the first occurrence of "score" refers to the cost sigma, and the second occurrence refers to E, but I'm not sure.

This section has been rewritten to remove the confusion. We now refer to a parsimony score and make explicit references to the values assigned to the E matrices without using the term score an additional time. One important passage is found on p. 26:

Edge choices that lead to the best parsimony score at the top-most branch point are traced back and recorded at each branch point *k* in **E**^(k)^; considering direction, **E**^(k)^ *(a, b)* = 1 if *(a,b)* forms part of the optimal trace; likewise, **E**^(k)^ *(b, a)* = 1 if *(b,a)* is chosen, utilising either side of the diagonal of the matrix.

** 1d. The whole delta = IN, OUT bit sounds very confusing to me. Couldn't the authors just write (a,b) for one direction and (b,a) for the other?

As highlighted above, the suggested notation has been adopted and is now referenced as such in the text, equations, and new Figure 6.

** 1e. The inference for a node k seems to be only based on the inferences for its descendants. This is very unusual, as most ASR algorithms include a bottom-up and a top-down phase, allowing information to flow in all directions within the input tree. Does this mean that to get the marginal reconstruction at a specific node, the tree must be re-rooted in that node? If that's the case, it must be said.

Definitely not. Equation 6 (previously Eq. 2) defines the probability of a state at *any* ancestor and *any* position (as long as that ancestor has character content at that position, i.e. not subject to indels) based on a multiplication across all conditional probabilities for *all* extant and ancestor branchpoints (again subject to indels) regardless of their relative position in the tree. The equation does not reference the children of the query ancestor, so we believe this is a simple misunderstanding or misreading of it.

For what it is worth, the implementation itself will involve the propagation of “factors” (by reference to algorithms like variable elimination in GRASP) across the topology in the most efficient manner. While some tools implement marginal reconstruction in a manner that involves re-rooting, it is *not* how GRASP implements it (which is by variable elimination, which is not a new algorithm, as cited in the paper).

**** (2) ****
The authors do not cite any paper from a whole line of work from the late 2000s about optimization problems for indel history reconstruction, including both maximum parsimony and maximum likelihood formulations. See for example the work in Mathieu Blanchette's group, the papers by Snir and Pachter (2006, 2011), and this PhD thesis for a review: <https://escholarship.mcgill.ca/concern/theses/xd07gv166>.

I do not consider this to be a major problem, because none of the algorithms developed in those works is likely to be applicable here, due to their poor scalability. Moreover, those works usually assume "phylogenetic correctness" of the ancestral reconstructions, that is, a character that has been deleted cannot be re-inserted back -- a requirement that (interestingly) is not imposed here.

Despite the important differences, I think that acknowledging and discussing some of these works is very important, to set the background.

We apologise for the omission. As remarked by the reviewer, there are issues with scalability that mean that we cannot directly apply this previous work, however we agree these studies should still be referenced as prior work of relevance.

As such, we add references in the Introduction to Diallo and colleagues, and Snir and Pachter’s work and in Methods, to explain issues of scalability of previous approaches and how the current study approaches this by decomposing the indel histories into multiple independent optimisations.

On p. 3:

insertion and deletion (indel) events introduce dependencies between positions in sequence that make their history difficult to access at greater scale (refs 12-13)

On p. 26 (integrated with the larger Methods rewrite at lines 610-611):

This inference involves solving multiple independent optimisation problems, avoiding scalability issues of earlier approaches (refs 12-13)

**** (3) ****
The authors appear to claim that GRASP produces *all* most parsimonious solutions (line 221). However they provide no proof, nor do they provide a clear definition of the parsimony cost function that they wish to optimize. (By the way, for Blanchette's definition of intel parsimony, the problem has been shown to be NP-hard by Chindelevitch et al. 2007, so any exact solution would be very surprising.)

While a formal treatment would probably be beyond the scope of this paper, I think that unproven claims should be avoided. Unless proven otherwise, the algorithms in GRASP are heuristic, and the authors should acknowledge this.

The reviewer was misled by our poor wording when discussing the difference between GRASP’s and FastML’s implementation of simple indel coding (SIC) inference: “We used parsimony inference to ensure that the implementations were comparable, noting that FastML only presents a single parsimonious indel history even when several are optimal, while GRASP presents all.”

The optimisation is for *one* indel at a time, not the whole sequence. For this level of optimisation, maximum parsimony commonly returns multiple solutions (that indel is present *or* absent). To present the ancestor in the form of a sequence, FastML must sometime choose either present *or* absent when both are optimal (for one indel), whereas a POG can represent both as alternative paths. GRASP collects *all* of the optimal states, presents them via a POG, and therefore never forces a choice. To be clear, there is no guarantee that the resulting sequence or POG is optimally parsimonious.

We changed the wording on p. 9:

We used parsimony inference to ensure that the implementations were comparable, noting that FastML presents a single state for each indel (independent of all other indels) as evident by the ancestor sequence. The flexibility of ancestor POGs enables GRASP to present both presence and absence of indel when both are deemed optimal (still independent of all other indels).

**Second sub-reviewer**:
In line 188, they stated the use of MAFFT to realign the sequences generated by INDELible. I have some experience aligning data coming from alternative splicing with big indels because of the changes in the intron-exon architecture. MAFFT is better than classical aligners, as it tends to create blocks, but it starts to generate errors in the alignments in some conditions, especially with high indel variability and homologous regions (exon duplications). In that context, I have found that ProGraphMSA is a lot better, as it tries to create a phylogenetically sensible gap pattern. I wonder whether such an aligner could better suit this study, but MAFFT is a good choice anyway. All this also makes me wonder how their method performs in the presence of homologous regions inside a protein; is it something challenging for the proposed algorithm?

Realigning the data within this context of the paper (the indel simulation section) was specifically to generate harder and more realistic test cases for different indel methods, given that they all performed similarly on the alignments generated by INDELible. To this end, absolute accuracy of the alignment method was not a crucial factor. Instead, we needed a consistent way to generate alignments where the true placement of gaps was not necessarily perfectly identified (as was the case in the known INDELible data).

The issue here is that any aligner is unlikely to return the exact alignment generated by INDELible, and so is unlikely to return accuracy scores comparable to the known data (since these accuracy measures are defined by specific start and end indexes of gaps based on the alignment). We did try realigning with ProGraphMSA but it did not offer improvements in this regard.

We also note and agree with the reviewer that MAFFT is a good choice to perform alignment and so believe it serves a good purpose within this section of the paper. That said, we certainly agree that is worthwhile to evaluate on aligners that may be better suited to this task by being phylogenetically-aware, and will incorporate ProGraphMSA as a choice worth evaluating in future research.

I have found it strange that they are mainly working with short indels. In line 954, they state that the maximum indel length created by INDELible is 10. Also, in line 633, they said that they had removed sequences that had the longest indel, over 20 amino acids. Therefore, I wonder how the algorithm performs in scenarios with longer indels.

There are a few points to be made here:

(a) The removal of indels over 20 amino acids is just for the CYP2U data set, where we are dealing with a single domain, longer indels (if not just extensions/trimming of the termini) are likely to be artefacts of low-quality sequences in the databases as these indels in actual sequences would most likely destabilise the fold. Changes in substrate specificity or catalytic efficiency could be affected by smaller indels, not just large ones, and are less likely to destabilise the structure, so would be as useful, if not more, for engineering purposes.

(b) Although INDELible is set to only introduce indels with a maximum length of 10, this still allows for indels to be introduced at neighbouring positions within the alignment and so, there is a cumulative effect of introducing deletions over simulated evolutionary time that can mean that although there is a maximum length for any given indel event, in practice these events can overlap and be represented as much longer contiguous gap positions.

I wonder why they avoid having indels from variations in the exon-intron architecture. They mention in line 637 that they have removed sequences that have a difference in exon number larger than two. Limiting the long indel can also help avoid significant changes in exon composition. Even that, I still wonder whether the CYP2U/2R/2D indel events could be related to alternative splicing or exon-intron architecture changes.

First, apologies for the lengthy response but this is an interesting remark, which we decided to look closely at; ultimately, the scope of the manuscript does not justify including an extended analysis but we still wanted to explain our reasoning.

For the first aspect of this question, it is worthwhile clarifying that the removal of sequences with alternative intron/exon architecture was a result of observing spurious, automatically annotated, sequences (with no experimental or cDNA data to verify) that had clear indications of completely non-conserved/non-homologous sequences that disrupt the core enzymatic fold of the protein. We reasoned that these sequences were, in most cases, likely to be due to poor quality sequence data, or sequences with miscalled intron/exon boundaries due to automatic annotation pipelines. A good example of this is the human gene for CYP2U1, where an alternative 7-exon structure is given by an automatic annotation pipeline (ENST00000508453.1) but has no experimental EST or cDNA data to verify its biological importance. By contrast, the 5-exon human CYP2U1 gene (ENST00000332884.11) is backed up by experimental reports and has been cloned and expressed as a functional cytochrome P450 enzyme. Finally, the 5-exon structure of the CYP2U1 and CYP2R1 subfamilies is a well-established feature in the literature (Nelson, 2004) and no evidence to date has indicated significant splice variants or isoforms for this gene in vertebrates – although it is possible that these do exist.

For the second aspect of the question, we also considered whether the CYP2U indels were a result of changes in the intron/exon boundary. Unlike the spurious changes discussed above, these indels were conserved across a number of species from the same linage, suggesting that these indels were genuine. As discussed below, there is a possibility that both indels could results from changes to the intron/exon boundaries but, given the existing data, we were unable to find sufficient evidence to either support or refute this hypothesis. We have amended the paper text accordingly to note this (see amended manuscript p. 12, lines 308-310):

Finally, we considered whether the indels in CYP2U ancestors resulted from changes to the intron/exon boundaries in the gene but were unable to find strong evidence either for or against this hypothesis.

To investigate the possibility that the CYP2U1 indels were generated from changes to the intron/exon architecture, instead of other mechanisms (e.g., double stranded DNA breaks), we examined the intron/exon boundaries of the human CYP2U1, CYP2R1 and CYP2D6 genes. Neither the AA’ loop insertion, nor the fish-specific insertion are near an intron/exon boundary in the human CYP2U1 sequence, suggesting that, if these indels resulted from changes to the intron/exon architecture, the evidence for this has been lost over the course of evolution. We consider that at this stage, other alternatives for this insertion (such as a double strand break repair by non-homologous end joining) are equally probable.

The fish insertion: Closer examination of the location of the fish insertion, does suggest a putative intron/exon boundary, which may have been implicated in this insertion in fish. However, this insertion is not directly adjacent to the intron/exon boundary in either the zebrafish CYP2U1 gene or the human CYP2U1 gene. Consequently, it is difficult to have great confidence in an intron-exon boundary event even for the fish insertion.

Response to Reviewers Fig 1: Location of the fish-specific insertion in the CYP2U1 genes from zebrafish and human.

The AA’ loop: In the human CYP2U1 gene, the AA’ loop (which is extended in the CYP2U proteins, relative to the closely related CYP2R and CYP2D subfamilies) is present in the middle of exon 1, which ends ~70 residues after the AA’ loop extension (see figure below). This would suggest that changes to the AA’ loop length in the *CYP2U* lineage did not result from changes to the intron/exon architecture.

Response to Reviewers Fig 2: The location of the AA’ loop extension in Exon 1 of human CYPU1.

Response to Reviewers Fig 3: The location of the CYP2U-specific AA’ loop extension in exon 1 of the human CYP2U1 gene, relative to the end of exon 1.

Of more interest is the origin of the AA’ loop in the CYP2U lineage, relative to the CYP2R and CYP2D subfamilies (see figure below). Here, there is some evidence that the AA’ loop may have resulted from a change in the intron/exon boundary during the early divergence of the CYP2U subfamily from the CYP2R and CYP2D subfamilies. Both CYP2R1 and CYP2D6 have much shorter sequences for exon 1 which, in these proteins, ends near the beginning of the AA’ loop extension in the CYP2U1 gene. Furthermore, the intron 1 for CYP2U1 is much longer than the intron 1 for CYP2R1.

Response to Reviewers Fig 4: The gene architecture of CYP2D6 and CYP2R1.

The divergence of the CYP2U and CYP2R subfamilies from the CYP2Ds and other vertebrate CYP2 subfamilies saw a change in the intron/exon architecture from 5 exons (CYP2U, CYP2R) to 9 exons (all other vertebrate CYP2 subfamilies) (Nelson, 2004). Therefore, it is possible that the AA’ loop extension seen in the CYP2Us did result from changes to the intron/exon boundary. However, only speculation is possible at present. In our view there is insufficient evidence to warrant the inclusion of such speculation in the manuscript. Furthermore, such issues, while of interest, are peripheral to the main concerns of the manuscript. Consequently, as mentioned above, we have noted this issue in the text (p. 12) but have not provided additional explanation or figures.

Response to Reviewers Fig 5: The intron 1 of hCYP2U1, hCYP2D6 and hCYP2R1

Also, they look to have edited their alignments so much that I wonder whether they have accessed biological insertion and deletions. For example, in line 503, they noted to have trimmed highly gapped columns and used Gblock. I guess that both things can change the number and length of indel events.

Only the GDH-GOx data set is trimmed using Gblocks, and only to infer a phylogenetic tree. The full alignment (except for a trimmed N- and C-termini is used to reconstruct the actual ancestors).

Additionally, the GDH-GOx reconstruction is not used to make any assertions about insertions and deletions, and all other data sets in the manuscript do not involve this filtering step.

We have added clarifying text to explain how Gblocks was used for the GDH-GOx data set (p. 33):

Marginal reconstruction of ancestral nodes was performed with the LG evolutionary rate model with indels inferred using bi-directional edge parsimony, after the N- and C-termini of the alignment had been trimmed but otherwise using the full alignment, not the alignment pruned by Gblocks.

In line 423, they talk about the possible structural location of the indels and their flexibility, but, sadly, the work doesn't have a structural analysis of the reconstructed sequences. I was expecting more on that front, but I guess they haven't seen anything interesting. In particular, because they have explicitly selected sequences with structural data available for the DHAD dataset.

We refer to our response to Reviewer 2 above (page 3 of this response letter).

In line 578, why haven't they subsampled the tree of all sequences to create the guide trees for the multiple sequence alignments avoiding having tree changes? In any case, I guess they would see the same trend but with more minor variations.

It would be possible to do as is suggested – create one large dataset / phylogenetic tree and to construct smaller trees by simply filtering out data not present in each of the step sizes. We were interested in creating a series of trees / alignments that were not necessarily subsets of each other in order to have each step size be an independent sample and to avoid potential bias from phylogenetic signal present in the largest data set.

This is in contrast to how the CYP2U1 data set is scaled up which, while not strictly a large tree that is subsampled, is built by adding data from sister clades, CYP2R and CYP2D. And so, in effect, the subtree containing all of the CYP2U1 sequences contains the exact same sequences, with potential clade-specific minor rearrangements and allows us to assess the effect of growing phylogenetic data sets in multiple manners.

Lastly, did you precisely understand how they filtered the MSA in line 719? The phrase wasn't clear to me.

We have changed the language to explain how Shannon entropy is calculated for each position and a summed value assigned to each sequence. We have also highlighted that this was not a drastic filtering step as we only reduce the data set from 11,920 sequences to 11,756 sequences. It is better thought of as a way to reproducibly remove the obvious outlier sequences that appear poorly aligned.

The following change was made on p. 40:

To objectively identify such outliers, we determined the proportion of gaps in each column, used this to calculate the Shannon entropy for a given sequence at each position and summed the entropies across each aligned sequence. This demarcated approximately the top 1% of sequences due to their atypical gap patterns, which we decided to remove, resulting in a final set of 11,756 sequences.

**Have the authors made all data and (if applicable) computational code underlying the findings in their manuscript fully available?**

Reviewer #1: Yes

Reviewer #2: **No:** As noted in Comments to Authors, the alignments and phylogenetic trees, in addition to resurrected sequences of ancestral proteins seem not to be found in the main text nor supplemental information as the forms which can be accessed by readers.

Reviewer #3: None

We apologise for not clearly indicating where data sets could be found. As indicated above in response to Reviewer 2, we have introduced a specific section in the manuscript entitled

Data availability on p. 33:

Data sets for CYP2U, DHAD, KARI, and GDH-GOx reconstructions are available from https://github.com/bodenlab/GRASP-resources. All different data sets within each family are included, representing a range of different sequence identities and sizes (Supplementary Fig. 16). Included are the original FASTA files, phylogenetic trees, sets of ancestors, and phylogenetic trees with labelled ancestral nodes. The full set of ancestors from each joint reconstruction is provided, except for GDH-GOx, where the single nominated marginal reconstruction at N320 is provided.

End of comments
